# ASR1 and ASR2, Two Closely Related ABA-Induced Serine-Rich Transcription Repressors, Function Redundantly to Regulate ABA Responses in Arabidopsis

**DOI:** 10.3390/plants12040852

**Published:** 2023-02-14

**Authors:** Hadia Hussain, Yuxin Cheng, Yating Wang, Yuan Yuan, Adnan, Yingying Li, Hainan Tian, Saddam Hussain, Siyu Chen, Rao Lin, Tianya Wang, Shucai Wang

**Affiliations:** 1 Key Laboratory of Molecular Epigenetics of MOE, Northeast Normal University, Changchun 130024, China; 2 Laboratory of Plant Molecular Genetics & Crop Gene Editing, School of Life Sciences, Linyi University, Linyi 276000, China

**Keywords:** ASR1, ASR2, CRISPR/Cas9, abscisic acid, transcription repressor, Arabidopsis

## Abstract

The plant hormone abscisic acid (ABA) is able to regulate the expression of ABA-responsive genes via signaling transduction, and thus plays an important role in regulating plant responses to abiotic stresses. Hence, characterization of unknown ABA response genes may enable us to identify novel regulators of ABA and abiotic stress responses. By using RT-PCR analysis, we found that the expression levels of *ABA-induced Serine-rich Repressor 1* (*ASR1*)and *ASR2*, two closely related unknown function genes, were increased in response to ABA treatment. Amino acid sequence analyses show that ASR1 contains an L×L×L motif and both ASR1 and ASR2 are enriched in serine. Transfection assays in Arabidopsis leaf protoplasts show that ASR1 and ASR2 were predominantly localized in the nucleus and were able to repress the expression of the reporter gene. The roles of ASRs in regulating ABA responses were examined by generating transgenic Arabidopsis plants over-expressing *ASR1* and *ASR2,* respectively, and CRISPR/Cas9 gene-edited single and double mutants for *ASR1* and *ASR2*. In both the seed germination and cotyledon greening assays, ABA sensitivity remained largely unchanged in the over-expression transgenic plants and the single mutants of *ASR1* and *ASR2*, but greatly increased ABA sensitivity was observed in the *asr1 asr2* double mutants. In root elongation assays, however, decreased ABA sensitivity was observed in the *35S*:*ASR1* and *35S*:*ASR2* transgenic plants, whereas increased ABA sensitivity was observed in the *asr1* and *asr2* single mutants, and ABA sensitivity was further increased in the *asr1 asr2* double mutants. Transcriptome analysis show that the differentially expressed genes (DEGs) down-regulated in the *35S*:*ASR1* transgenic plant seedlings, but up-regulated in the *asr1 asr2* double mutant seedlings were highly enriched in processes including responses to plant hormones and stress stimuli. Taken together, our results show that *ASR1* and *ASR2* are closely related ABA response genes, ASR1 and ASR2 are serine-rich novel transcription repressors, and they negatively regulate ABA responses in Arabidopsis in a redundant manner.

## 1. Introduction

Transcription repression mediated by the ERF-associated amphiphilic repression (EAR) motif-containing proteins has been considered to be the main form of transcriptional repression in plants [1]. EAR proteins are able to repress the expression of the downstream genes either via acting alone as transcription repressors or by recruiting co-repressors, therefore, to regulate plant growth and development, as well as plant responses to plant hormones and environmental stimuli [1,2,3,4,5,6,7,8,9]. For examples, ovate family protein 1 (OFP1) regulates cell elongation in Arabidopsis via acting as a transcription repressor to suppress the expression of *GA20-oxidase 1* (*GA20ox1*), a key biosynthesis gene of plant hormone Gibberellin (GA) [10], whereas KINASE-INDUCIBLE DOMAIN INTERACTING 8 (KIX8) and KIX9 regulate leaf growth in Arabidopsis via regulating the expression of downstream genes by recruiting the co-repressor TOPLESS [11].

The EAR motif was first identified from the class II ethylene responsive factor (ERF) repressors as an (L/F)DLN(L/F)×P amino acid signature [12], but was further refined to DLN××P and L×L×L motifs by analyzing the class II ERFs, C2H2 transcription factor repressors, and some other EAR proteins [1]. A genome-wide identification of EAR proteins by searching the L×L×L or DLN××P EAR motifs in protein amino acid sequences revealed that there are more than 400 genes in Arabidopsis encoding EAR proteins, and most of them are functionally uncharacterized [7]. On the other hand, EAR proteins identified so far showed a high diversity on their amino acid sequences [7], therefore it is very likely that some EAR proteins remain unidentified. As evidences, some of the ABA-induced transcription repressors (AITRs) contain an L×L×L motif but were not previously identified in the genome-wide identification, and we found that they are involved in the regulation of plant responses to plant hormone abscisic acid (ABA) and abiotic stresses [13,14]. On the other hand, we also found that *Arabidopsis thaliana* EAR motif-containing ABA up-regulated 1 (AtEAU1) and AtEAU2, two functionally uncharacterized EAR proteins previously identified in the genome-wide identification, are involved in the regulation of ABA responses [9].

ABA is a key plant hormone in regulating plant abiotic stress responses [15,16,17]. Abiotic stresses such as drought and salt stimuli promote ABA accumulation [18,19]. ABA molecules, in turn, bind to the pyrabactin resistance/PYR1 like/regulatory components of ABA receptors (PYR/PYL/RCAR) [20,21], and therefore promote the binding of PYR/PYL/RCAR receptors with the type 2C protein phosphatases (PP2Cs), the ABA signal negative regulators [22,23], leading to the release from the inhibition of PP2Cs, and self-activation of the protein kinases Snf1 (sucrose-non-fermentation 1)-related kinases subfamily 2 (SnRK2s), the ABA signal positive regulators [24]. The self-activated SnRK2 kinases are able to phosphorylate the downstream ABF/AREB/ABI5-type bZIP transcription factors [25,26], which are then able to activate or repress the expression of downstream ABA response genes, thereby affecting plant responses to abiotic stresses [15,16,26,27,28,29,30,31].

Regulators encoded by some of the ABA response genes, such as the B3 transcription factor ABI3, the APETALA2 (AP2) transcription factor ABI4, the bZIP transcription factor gene AtbZIP62, and the R2R3 MYB transcription factor MYB71, in turn, are able to regulate ABA response in Arabidopsis, thereby providing feedback regulation loops of ABA signaling [32,33,34,35]. By characterizing unknown function ABA response genes, we have previously identified some novel regulators of ABA and abiotic stress responses, and some of them, including a few AITRs and the AtEAUs, are EAR proteins as mentioned above [9,13].

Here we report the identification and characterization of ABA-induced Serine-rich Repressor 1 (ASR1) and ASR2, two closely related novel regulators of ABA responses from unknown function ABA response genes. We found that only ASR1 contains an L×L×L EAR motif, but both ASR1 and ASR2 are enriched in serine, a feature observed in the middle region (MR) of the auxin response factor (ARF) repressors [36,37], and function as transcription repressors. Assays with the over-expression transgenic plants and gene-edited mutants obtained show that ASR1 and ASR2 negatively regulate ABA responses in Arabidopsis in a redundant manner.

## 2. Results

### 2.1. ASR1 and ASR2 Are Closely Related ABA-Induced Serine-Rich Protein Genes

**Figure 1:**
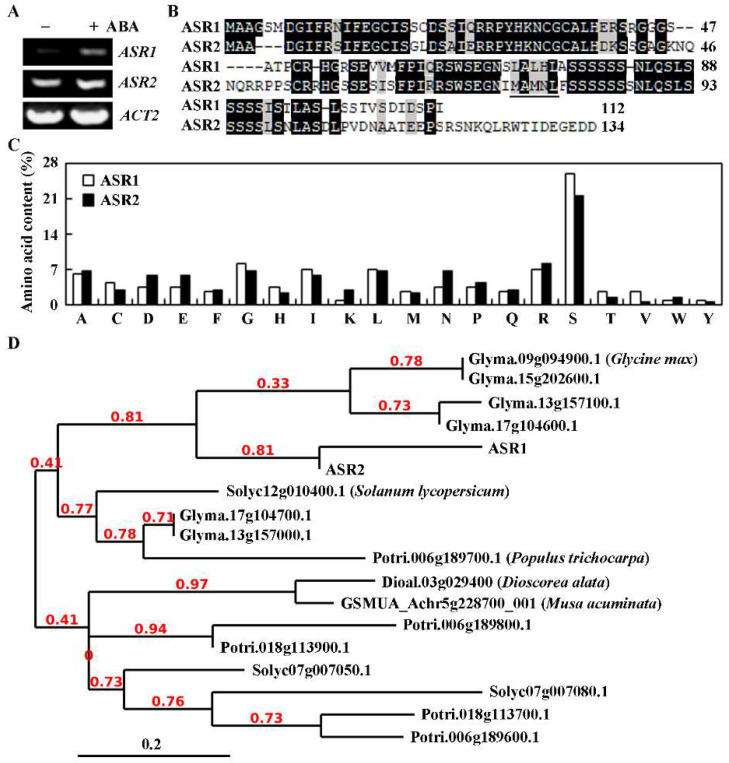
Expression of *ASR1* and *ASR2* in response to ABA treatment and amino acid sequence analysis of ASR1 and ASR2. (**A**) Effects of ABA on the expression of *ASR1* and *ASR2.* Ten-day-old seedlings of the Col wild-type Arabidopsis were treated with 50 μM ABA for 4 h, RNA was isolated, and RT-PCR was used to examine the expression of *ASR1* and *ASR2*. *ACT2* was used as a control. (**B**) Amino acid sequence alignment of ASR1 and ASR2. Full length amino acid sequences were subjected to sequence alignment by using BioEdit. The identical amino acids were shaded in black, and the similar ones in gray. Under line indicates the L×L×L motif in ASR1. (**C**) The percentage of amino acid content of ASR1 and ASR2. Amino acid content was analysis on NovoPro (https://www.novopro.cn/tools/protein_stats.html, accessed on 25 December 2022). (**D**) Phylogenetic analysis of ASRs and their homologues from several different plants. Full-length amino acid of ASRs and their homologs were subjected to phylogenetic analysis by using “one click” mode with default settings on phylogeny (https://www.phylogeny.fr/, accessed on 7 February 2023).

In the process of identification and characterization of novel regulators of ABA and/or abiotic stress responses from unknown function ABA response genes as described before [13], we found that At5g35110 is an unknown function ABA response gene in the transcriptome dataset. To examine if the expression of At5g35110 is indeed induced by ABA, we compared its expression levels in ABA-treated and mock-treated Col wild-type Arabidopsis seedlings. The seedlings were treated with ABA or mock treated with solvent alone for 4 h, RNA was isolated, and the expression level of At5g35110 was determined by RT-PCR. As shown in Figure 1A, the expression level of At5g35110 was greatly increased in ABA-treated seedlings when compared to the mock-treated seedlings. As At2g46490 is the only closely related gene to At5g35110, we also examined its expression in response to ABA, and we found that its expression level was also increased in response to ABA treatment (Figure 1A).

By examining the amino acid sequences, we found that proteins encoded by both genes are enriched in the amino acid serine (Figure 1B), the content of which is more than 20% of the total amino acids in both proteins (Figure 1C). In addition, both proteins showed transcription repression activities in transfected protoplasts (see next section for details), and we therefore named At5g35110 and At2g46490 genes *ABA-induced Serine-rich Repressor 1* (*ASR1*) and *ASR2*, respectively.

Homologs of ASRs are widely distributed in different plant species (https://phytozome-next.jgi.doe.gov/, accessed on 5 February 2023). Full-length amino acids of homologs from several different plants, including the eudicot plants tomato (*Solanum lucopersicum*), soybean (*Glycine max*) and poplar (*Populustrichocarpa*), and the monocot plants purple yam (*Dioscoreaalata*) and banana (*Musa acuminata*), were obtained (File S1), and subjected to phylogenetic analysis with ASR1 and ASR2. The results show that ASR1 and ASR2 are closely related, and some homologs from the selected eudicot and monocot plants are clustered in the same clade (Figure 1D).

### 2.2. ASR1 and ASR2 Are Transcription Repressors

ARFs proteins with a serine-rich MR are transcription repressors [36,37], considering that both ASR1 and ASR2 are enriched in serine, and ASR1 also contains an L×L×L EAR motif (Figure 1B), even though it is was not identified as an EAR motif-containing protein in previously assay [7], suggesting that ASR1 and ASR2 may function as transcription repressors.

**Figure 2:**
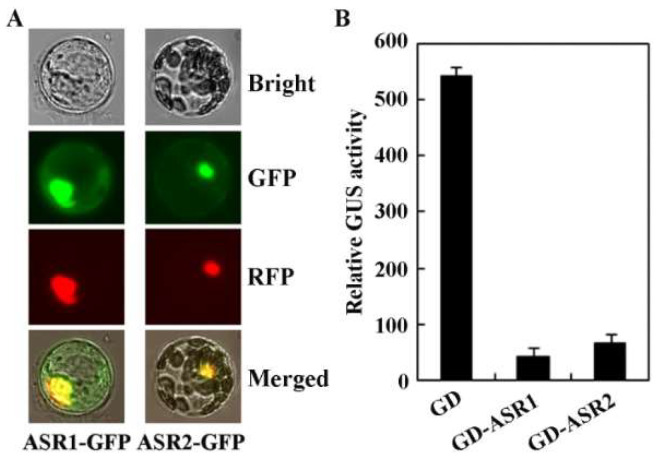
Sub-cellular localization and transcriptional activities of ASR1 and ASR2. (**A**) Sub-cellular localization of ASR1 and ASR2. Plasmids of *ASR1-GFP* and *ASR2-GFP* were co-transfected, respectively, with the nuclear marker gene *NLS-RFP* into isolated Arabidopsis protoplasts, the transfected protoplasts were incubated for 18–22 h under darkness at room temperature, then the GFP and RFP florescence was observed under a florescence microscope and pictures were taken. (**B**) Transcriptional activities of ASR1 and ASR2. Plasmids of *GD-ASR1* and *GD-ASR2* were co-transfected, respectively, with *LD-VP* activator and the *LexA-Gal4*:*GUS* reporter constructs into isolated Arabidopsis protoplasts, the transfected protoplasts were incubated for 18–22 h under darkness at room temperature, then GUS activity was analyzed by using a microplate reader. The *GD* plasmid was co-transfected as a control. The data represent the mean ± SD of three repeats.

We therefore examined the transcription activities of ASR1 and ASR2 by using transient transfection in Arabidopsis leaf protoplasts. We first examined the sub-cellular localization of ASR1 and ASR2. Plasmids of the *ASR1-GFP* and *ASR2-GFP* constructs were co-transfected, respectively, with plasmids of the nuclear marker construct *NLS-RFP* [38], into isolated Arabidopsis protoplasts, and GFP and RFP fluorescence was examined under a fluorescence microscope after overnight incubation of the transfected protoplasts. As shown in Figure 2A, GFP fluorescence was predominantly overlapped with RFP fluorescence, indicating that ASR1 and ASR2 are localized in the nucleus.

We then examined transcriptional activities of ASR1 and ASR2 by using a protoplast transfection system designed to examine transcription repression activities [39]. In this system, plasmids of *LexA-Gal4*:*GUS*, the reporter construct, were co-transfected with *LD-VP*, the activator construct, and the GD-fused *ASR1* or *ASR2* effector construct or the *GD* control construct into Arabidopsis protoplasts. As a result, LD-VP is able to activate the reporter gene, and the activities are reduced if the co-transfected effector functions as a transcription repressor. As shown in Figure 2B, activities of the reporter gene activated by LD-VP were reduced in protoplasts co-transfected with *GD-ASR1* or *GD-ASR2*, indicating that both ASR1 and ASR2 function as transcription repressors.

### 2.3. Generation of Overexpression Transgenic Plants and Gene Edited Mutants for ASR1 and ASR2

ASR1 and ASR2 were identified during the identification and characterization of novel regulators of ABA and/or abiotic stress responses; therefore, we wanted to further examine the functions of ASR1 and ASR2 after showing that they are transcription repressors. To accomplish that, we generated transgenic over-expression plants and CRISPR/Cas9 gene-edited mutants for *ASR1* and *ASR2.*

Transgenic plants over-expressing *ASR1* and *ASR2* were generated by transforming the Col wild-type Arabidopsis plants with *pPZP211-35S*:*ASR1* and *pPZP211-35S*:*ASR2,* respectively, and selecting homozygous over-expression plants in the T3 generation. Two independent over-expression lines for each of the genes, i.e., *35S*:*ASR1* #10 and #19, and *35S*:*ASR2* #21 and #22, were isolated and used in the following experiments.

**Figure 3:**
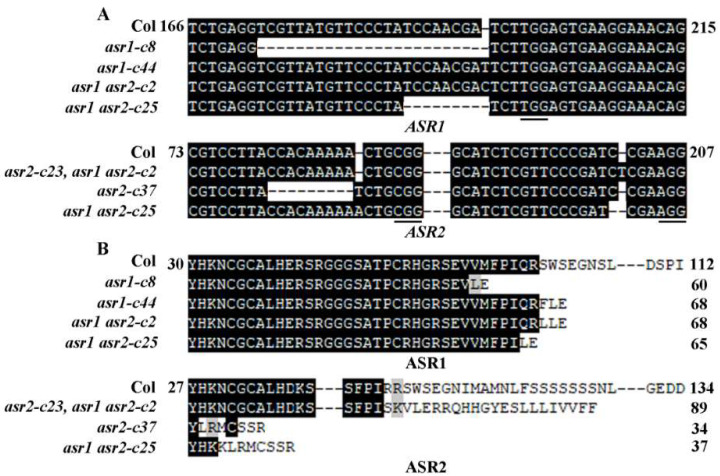
Generation of gene-edited mutants for *ASR1* and *ASR2*. (**A**) Editing status of *ASR1* and *ASR2* in the *asr1* and *asr2* single and the *asr1 asr2* double mutants. DNA was extracted from leaves of normal flowering T2 plants and used for RT-PCR amplification of *ASR1* and *ASR2*. The PCR products were sequenced, and the sequences obtained were aligned with the *ASR1* and *ASR2* sequences from the Col wild-type plants. Underlines indicate the PAM sites, and numbers indicate the position of the nucleotide relative to the first nucleotide of the wild-type *ASR1* and *ASR2* ORFs. (**B**) Amino acid alignment of ASR1 and ASR2 in the Col wild type, the *asr1* and *asr2* single and the *asr1 asr2* double mutants. The ORFs of *ASR1* and *ASR2* in the *asr1* and *asr2* single and the *asr1 asr2* double mutants were identified by using ORF finder (https://www.ncbi.nlm.nih.gov/orffinder/, accessed on 31 January 2022), and corresponding amino acids were then used for sequence alignment. Numbers indicate the position of the amino acid relative to the first amino acid of the wild-type ASR1 and ASR2 proteins.

CRISPR/Cas9 gene-edited single mutants for *ASR1* and *ASR2* were generated by transforming Col wild-type Arabidopsis plants with the *pHEE-FT* constructs containing two target sequences to *ASR1* or *ASR2*, respectively, and selecting Cas9-free homozygous mutants in the T2 generation. Two single mutants for each gene, i.e., *asr1-c8*, and *-c44*, and *asr2-c23* and *-c37*, were isolated and used in the experiments. Two CRISPR/Cas9 gene-edited double mutants, i.e., *asr1 asr2-c2* and -*c25*, were also generated. The *asr1 asr2-c25* double mutant was generated in the same way as for the single mutants but the *pHEE-FT* constructs used for plant transformation contains two target sequences to *ASR1* and two targets to *ASR2*, whereas the *asr1 asr2-c2* double mutant was generated by transforming the *asr2-c23* single mutant with the *pHEE-FT* construct containing two target sequences to *ASR1*. In all the mutants, either a single nucleotide insertion, or one to a few nucleotides deletion occurred in at least one target site (Figure 3A), resulting in several amino acids substitutions and a premature stop in ASR1 and ASR2, respectively (Figure 3B).

### 2.4. ASR1 and ASR2 Function Redundantly to Regulate ABA Responses in Arabidopsis

By using the over-expression transgenic plants and the mutants generated for *ASR1* and *ASR2*, we examined the functions of ASR1 and ASR2 in regulating ABA responses in Arabidopsis by using three typical assays, i.e., ABA inhibited seed germination, cotyledon greening, and root elongation.

**Figure 4:**
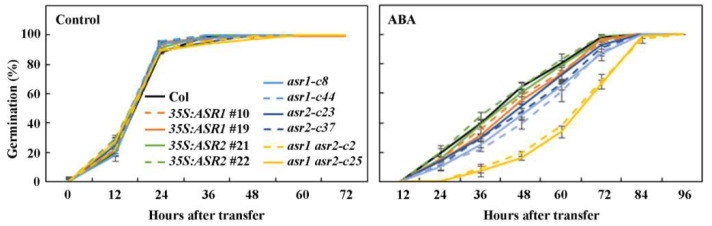
Effects of ABA on seed germination of the Col wild type, the *35S*:*ASR1* and *35S*:*ASR2* transgenic plants, the *asr1* and *asr2* single and the *asr1 asr2* double mutants. Sterilized seeds were sown on 1/2 MS plates with or without 0.5 μM ABA. The plates were kept at 4 °C in dark for 2 days and then transferred to a growth room. Germinated seeds were counted every 12 h after the transfer until all the seeds were germinated, and percentage of germination was calculated. Data represent the mean ± SD of three repeats.

In the seed germination assays, we found that in the control plates, almost all the seeds of the different plants, including the Col wild type, the *35S*:*ASR1* and *35S*:*ASR2* transgenic plants, the *asr1* and the *asr2* single and the *asr1 asr2* double mutants germinated 24 h after the plates were transferred from 4 °C to the growth room (Figure 4).

In the presence of ABA, germination was delayed, but no big difference was observed for the Col wild type, the *35S*:*ASR1* and the *35S*:*ASR2* transgenic plants, and the *asr1* and the *asr2* single mutants, however, greatly lower germination rates were observed for the *asr1 asr2* double mutants (Figure 4).

**Figure 5:**
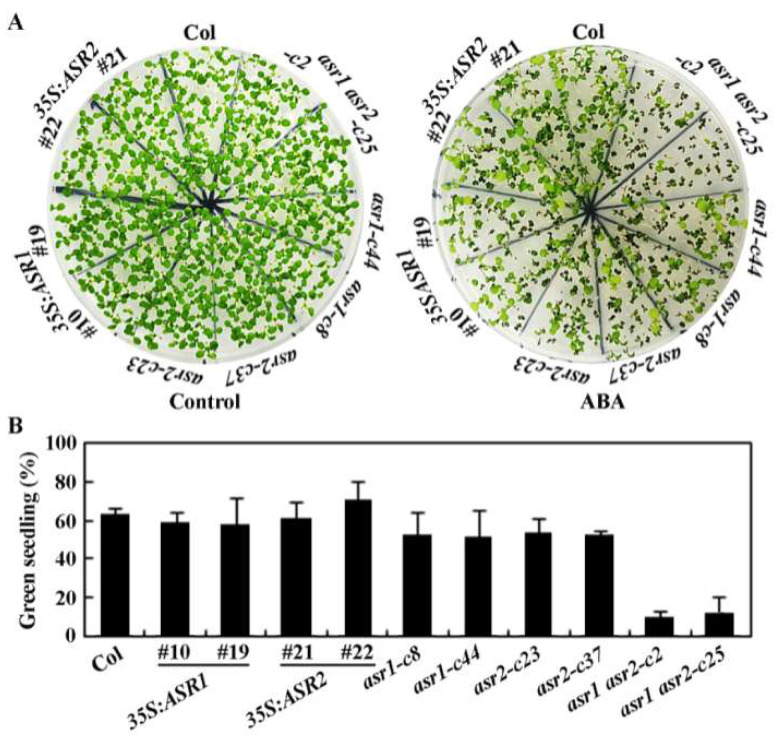
Effects of ABA on cotyledon greening of the Col wild type, the *35S*:*ASR1* and *35S*:*ASR2* transgenic plants, the *asr1* and *asr2* single and the *asr1 asr2* double mutants. (**A**) Cotyledon greening of the Col wild type, the *35S*:*ASR1* and *35S*:*ASR2* transgenic plants, the *asr1* and *asr2* single and the *asr1 asr2* double mutants in response to ABA treatment. Sterilized seeds were sown on 1/2 MS plates with or without 0.5 μM ABA. The plates were kept at 4 °C in dark for 2 days and then transferred to growth room. The numbers of seedling with green cotyledons were counted and pictures were taken 12 days after the transfer. (**B**) Percentage of green seedlings of the Col wild type, the *35S*:*ASR1* and *35S*:*ASR2* transgenic plants, the *asr1* and *asr2* single and the *asr1 asr2* double mutants in response to ABA treatment. Numbers of seedlings with green cotyledons were counted and percentage of green seedlings was calculated. Data represent the mean ± SD of three replicates.

Similar results were observed in the cotyledon greening assays, i.e., in the absence of ABA, almost all the seedlings for the plants including the Col wild type, the *35S*:*ASR1* and the *35S*:*ASR2* transgenic plants, the *asr1* and the *asr2* single and the *asr1 asr2* double mutants turned to green, but in the presence of ABA, a difference was clearly observed between the *asr1 asr2* double mutants and other plants (Figure 5A).

Quantitative analysis shows that the cotyledon greening rate for the Col wild type, the *35S*:*ASR1* and the *35S*:*ASR2* transgenic plants, and the *asr1* and the *asr2* single mutants, was ~60% at the present of ABA, whereas that for the *asr1 asr2* double mutants was only ~20% (Figure 5B).

**Figure 6:**
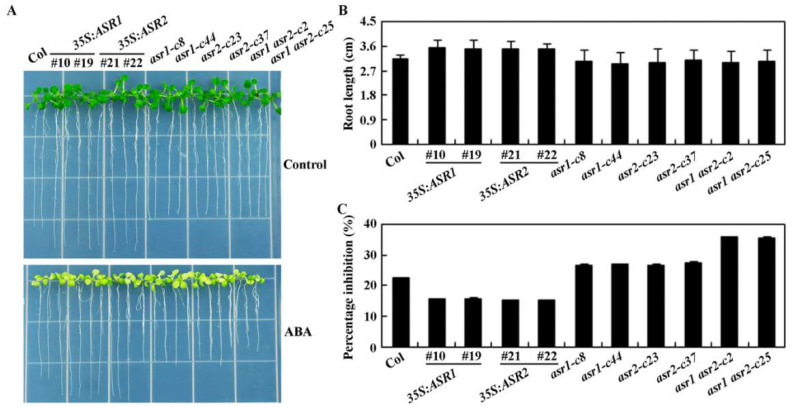
Primer root length and effects of ABA on root elongation of the Col wild type, the *35S*:*ASR1* and *35S*:*ASR2* transgenic plants, the *asr1* and *asr2* single and the *asr1 asr2* double mutants. (**A**) Seedlings of the Col wild type, the *35S*:*ASR1* and *35S*:*ASR2* transgenic plants, the *asr1* and *asr2* single and the *asr1 asr2* double mutants on plates with or without ABA. Sterilized seeds of the Col wild type, the *35S*:*ASR1* and *35S*:*ASR2* transgenic plants, the *asr1* and *asr2* single and the *asr1 asr2* double mutants were sown on 1/2 MS plates and kept at 4 °C in dark for 2 days, then transferred to a growth room and placed vertically for 4 days. The seedlings were then transferred to new plates with or without 10 μM ABA, and grown vertically for an additional 10 days, and then pictures were taken. (**B**) Quantitative analysis of the primary root length of the Col wild type, the *35S*:*ASR1* and *35S*:*ASR2* transgenic plants, the *asr1* and *asr2* single and the *asr1 asr2* double mutants. Root length on plates without ABA were measured and calculated. Data represent the means ± SD of 20 seedlings. (**C**) Inhibition of root elongation by ABA. Length of new elongated roots on plates with or without ABA was measured, and percentage of inhibition was calculated. Data represent the means ± SD of 20 seedlings.

In the root elongation assays, however, a difference in ABA response was clearly observed for both the over-expression transgenic plants and the mutants as compared with the Col wild type plants, and both the *35S*:*ASR1* and the *35S*:*ASR2* transgenic plant seedlings produce relatively longer roots in the absence of ABA as compared to the Col wild-type plant seedlings, whereas that of the single and double mutants was largely similar to the Col wild type (Figure 6A). Quantitative analysis further confirmed the observation, as shown in Figure 6B, the root length for both the *asr1* and the *asr2* single and the *asr1 asr2* double mutants was ~3.0 cm, similar to that of the Col wild type seedlings, whereas that of the *35S*:*ASR1* and the *35S*:*ASR2* transgenic plant seedlings was ~3.5 cm.

In the presence of ABA, root elongation was inhibited for all the seedlings, but to different degrees; an ~22% inhibition was observed for the Col wild type plant seedlings, ~15% for both of the *35S*:*ASR1* and the *35S*:*ASR2* transgenic plant seedlings, ~27% for the *asr1* and the *asr2* single mutants, and ~36% for the *asr1 asr2* double mutant seedlings (Figure 6C). These results indicate that ABA sensitive was decreased in the *35S*:*ASR1* and the *35S*:*ASR2* transgenic plants, but increased in the *asr1* and the *asr2* single mutants, and further increased in the *asr1 asr2* double mutants.

### 2.5. ASR1 and ASR2 Regulated Genes Are Enriched in Responses to Development, Hormone and Stress Stimuli

To explore the function mechanism of ASR1 and ASR2, we compared gene expression in the Col wild type, the *35S*:*ASR1* transgenic plants and the *asr1 asr2* double mutants by using transcriptome analysis. As ASR1 and ASR2 function as transcription repressors (Figure 2), we focused on the genes whose expression levels were decreased in the *35S*:*ASR1* transgenic plants but increased in the *asr1 asr2* double mutants.

**Figure 7:**
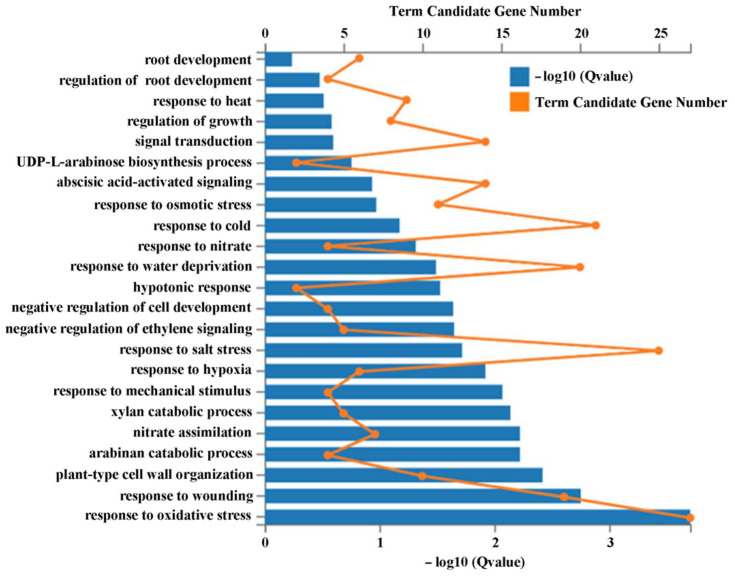
Functional classification of the DEGs. The DEGs that were down-regulated in the *35S*:*ASR1* #19 transgenic plants and up-regulated in the *asr1 asr2–c25* double mutants were subjected to functional gene ontology (GO) under the term of biological process. The GO annotation enrichment of DEGs analysis was evaluated using the phyper function in the R program. Significant enrichment was defined as GO keywords with a Q value (adjusted *p* value) < 0.05.

A total of 130 different expressed genes (DEGs) were found with reduced expression levels in the *35S*:*ASR1* transgenic plants but increased expression levels in the *asr1 asr2* double mutants (Table S1). Gene ontology (GO) analysis shows that DEGs were enriched in several different processes such as development, including root development, cell development and cell wall organization, metabolic processes including UDP-L-arabinose biosynthesis, xylan catabolic and arabinan carbolic, and nitrate response and assimilation (Figure 7). However, the DEGs were mostly enriched in responses to hormones, including ABA and ethylene, and stress stimuli including heat, osmotic, cold, salt, hypoxia, wounding, and oxidative stress (Figure 7).

**Figure 8:**
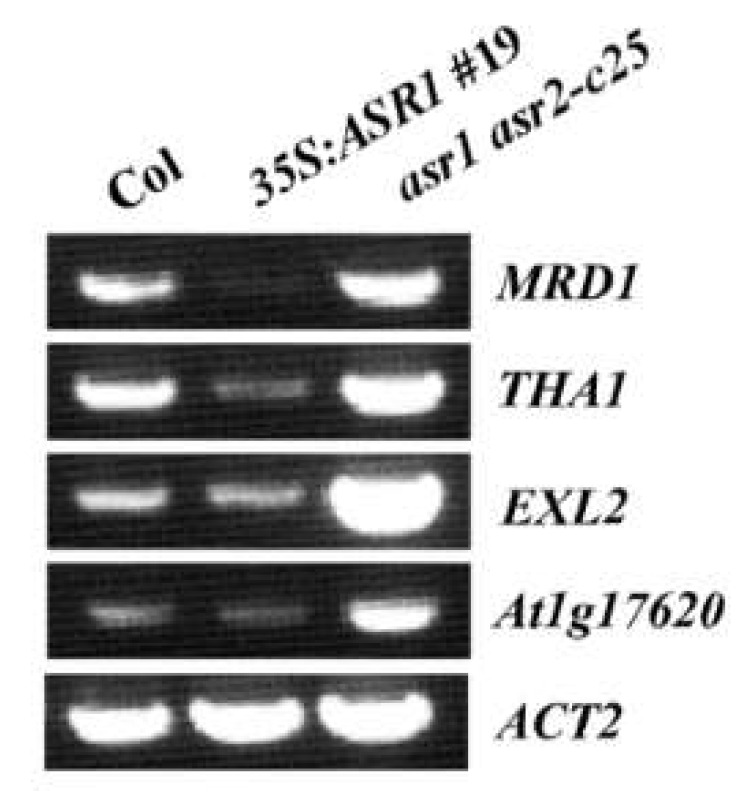
RT-PCR analysis of selected DEGs in the *35S*:*ASR1* transgenic plants and the *asr1 asr2* double mutants. RNA was isolated from 10-day-old seedlings of the Col wild type, the *35S*:*ASR1*#19 transgenic plants and the *asr1 asr2-c25* double mutants and RT-PCR was used to examine the expression of selected DEGs. *ACT2* was used as a control.

Among the DEGs with decreased expression levels in the *35S*:*ASR1* transgenic plants but increased expression levels in the *asr1 asr2* double mutants, several genes including *MTO 1 Responding Down 1* (*MRD1*), *Threonine Aldolase 1* (*THA1*), *Exordium Like 2* (*EXL2*), and *At1g17620*, a Late Embryogenesis Abundant (LEA) gene, have previously been shown to be induced by ABA, or involved in the regulation of ABA responses [40,41,42,43]. We therefore further analyzed their expression by RT-PCR, and the results show that their expression levels were indeed decreased in the *35S*:*ASR1* transgenic plants and increased in the *asr1 asr2* double mutants (Figure 8), consistent with the transcriptome analysis results (Table S1).

## 3. Discussion

EAR motifs are the repression motifs presented in transcription repressors such as Class II ERFs, some C2H2 transcription factors, and Aux/IAA proteins [1,7,12,39], and EAR protein-mediated transcription repression has been considered to be the main form of transcriptional repression in plants [1]. In addition, some ARFs with a serine-rich MR have also been shown to function as transcription repressors [36,37]. We provide evidence in this study that ASR1 and ASR2 are two novel transcription factors enriched in serine, and they play a redundant role in regulating ABA responses in Arabidopsis.

First, evidence suggests that both ASR1 and ASR2 function as transcription repressors. Even though both proteins shared high amino acid sequence identity and similarity, ASR1 contains a typical L×L×L EAR motif, but ASR2 does not (Figure 1). However, both ASR1 and ASR2 are enriched in serine (Figure 1), similar to that observed in the MR of ARF repressors [36,37]. These features indicated that ASR1 and ASR2 may function as transcription repressors. Indeed, both ASR1 and ASR2 mainly localized in the nucleus, and they repressed the expression of the reporter gene in transfected protoplasts when recruited to the promoter region by a fused DNA binding domain (Figure 2). In our previously research, we have shown that OFP1 and the AITRs with the L×L×L motif removed still function as transcription repressors, but with decrease transcription repression activities [10,13]. Similar, we found here that even though ASR2 does not contain an L×L×L EAR motif, it still functions as a transcription repressor, and its repression activities only reduced a little bit as compared with that of ASR1 (Figure 2). Since both ASR1 and ASR2 are enriched in serine (Figure 1), and ARFs with serine-rich MR function as transcription repressor [36,37], it is very likely that enriched serine in ASR1 and ASR2 contributed a major portion to their transcription repression activities. Yet protoplast transfection assays with ASR1 with the L×L×L EAR motif mutated will provide additional information if the L×L×L EAR motif is responsible for the slightly increased repression activities observed in ASR1.

Second, we found that both *ASR1* and *ASR2* are ABA responsive genes, and they are involved in the regulation of ABA responses in Arabidopsis. Our RT-PCR show that the expression levels of both *ASR1* and *ASR2* increased in response to ABA treatment, but to a different degree, i.e., the expression level of *ASR1* to ABA treatment increased to a relatively higher level when compared with that of*ASR2* (Figure 1). However, both ASR1 and ASR2 are negative regulators, and they function redundantly in the regulation of ABA responses in Arabidopsis as dramatically increased ABA sensitively was observed in the *asr1 asr2* double mutants in seed germination (Figure 4), seedling greening (Figure 5), as well as root elongation assays (Figure 6). Yet some difference was observed in different assays. In both seed germination and seedling greening assays, ABA responses of all the over-expression transgenic plants and the single mutants are largely indistinguishable from that of the Col wild-type plants (Figure 4 and Figure 5), whereas in root elongation assays, decreased ABA sensitivity was observed in the over-expression transgenic plants, and increased ABA sensitivity was observed in the single mutants, possibly because ABA sensitivity may be different in different growth stages, and/or due to the different concentrations of ABA used for different assays. Considering that different changes in ABA sensitivity were observed in the over-expression transgenic plants and the mutants for some of the novel regulator genes we previously identified [9,13], and root length was increased in the over-expression transgenic plants, it will be of interest to examine if differences observed in the root elongation assays were due to the function of ASR1 and ASR2 in regulating root elongation.

Third, we found that ASR1 and ASR2 may regulate the expression of genes related to plant hormone signaling and abiotic stress responses. As both ASR1 and ASR2 function as transcription repressors (Figure 2), and have redundant function in regulating ABA responses (Figure 4, Figure 5 and Figure 6), we identified genes with expression levels decreased in seedlings of the transgenic plant over-expressing *ASR1*, but increased in the *asr1 asr2* double mutant seedlings by using transcriptome sequencing. We found that a big portion of the 130 DEGs identified are enriched in responses to hormones (Figure 7), and RT-PCR results of several selected genes that respond to ABA or are involved in the regulation of ABA responses are consistent with the transcriptome assays (Figure 8), indicating that ASR1 and ASR2 may regulate ABA response in Arabidopsis via regulating the expression of ABA response-related genes. Considering that expression changes of ABA response genes will eventually lead to changes in plant responses to abiotic stresses [15,26,27,28,29,30,31], ABA sensitivity was altered in the over-expression transgenic plants and mutants of *ASR1* and *ASR2* (Figure 4, Figure 5 and Figure 6), and a big portion of the 130 DEGs identified as enriched in responses to abiotic stress stimuli (Figure 7); it is very likely that ASR1 and ASR2 may also be involved in the regulation of plant responses to abiotic stresses, yet experimental data are required to examine if this is indeed the case.

Considering that ASR1 and ASR2 are two closely related novel transcription repressors, it will be of great interest to examine if they can function alone, or act only as co-repressors in regulating target gene expression. It will be also of great interest to examine if any of the 130 DEGs are directly target genes of ASR1 and/or ASR2.

## 4. Materials and Methods

### 4.1. Plant Materials and Growth Conditions

The Columbia-0 (Col) ecotype of Arabidopsis (*Arabidopsis thaliana*) stored in our lab was used for plant transformation to produce transgenic plants and gene-edited mutants, for protoplast isolation for transfection assays, for examining ABA response of *ASR1* and *ASR2*, and as a control for ABA sensitivity assays and transcriptome sequencing.

To grow plants for plant transformation and protoplast isolation, seeds of the Col wild type were soaked in sterile water and kept at 4 °C in darkness for 2–3 days, and then sown directly into soil pots and kept in a growth room under a 16/8 h light/dark cycle with light density at ~120 μmol m^−2^s^−1^ and the temperature at 22 °C.

To grow seedlings for RNA isolation and ABA sensitivity assays, sterilized seeds of the Col wild type, the *35S*:*ASR1* and *35S*:*ASR2* transgenic plants, the *asr1* and *asr2* single and the *asr1 asr2* double mutants were germinated and grown in 1/2 Murashige and Skoog (MS) plates in a growth room.

### 4.2. ABA Treatment, RNA Isolation and RT-PCR

To examine the ABA response of *ASR1* and *ASR2*, 10-day-old Col wild-type seedlings grown in 1/2 MS plates were collected, and treated with 50 µM ABA solution or solvent methanol alone as a control for 4 h by shaking in darkness. The treated seedlings were then frozen in liquid nitrogen and used for RNA isolation.

To examine the expression level of *ASR1* and *ASR2* in the over-expression transgenic plants, 10-day-old seedlings of the homozygous transgenic plants grown in 1/2 MS plates were collected, frozen in liquid nitrogen and used for RNA isolation.

To compare the expression levels of *MRD1*, *THA1*, *EXL2,* and *AT1G17620*, four selected differentially expressed genes (DEGs) in the Col wild type, the *35S*:*ASR1* transgenic plants and the *asr1 asr2* double mutants, 10-day-old seedlings of the Col wild type, the *35S*:*ASR1*#19 transgenic plant, and the *asr1 asr2-c25* double mutant grown in 1/2 MS plates were collected, frozen in liquid nitrogen, and used for RNA extraction.

RNA isolation and cDNA synthesis were performed as reported previously [44]. Briefly, total RNA was isolated from the samples by using the EasyPure Plant RNA Kit (TransGene Biotech, Beijing, China), and cDNA was synthesized by using the EasyScript First Strand DNA Synthesis Super Mix (TransGen Biotech, Beijing, China) according to the instructions provided by manufacturer.

The RT-PCR cycles used for amplifying *ASR1* and *ASR2* were 29, and 25 for *ACT2* control with an annealing temperature of 52 °C, and those for *MRD1*, *THA1*, *EXL2* and *At1g17620* were 32 with 54 °C. The primers used for the amplification of *ASR1* were 5′-CAACATATGGCTGCCGGATCCATGGA-3′ and 5′-CAAGAGCTCTTAAATGGGAGAATCAATA-3′, for *ASR2* were 5′-CAACATATGGCCGCCGACGGAATATT-3′ and 5′-CAAGAGCTCTCAATCATCTTCACCTTCAT-3′, for *MRD1* were 5′-ATGAACTCAACTCAACAGT-3′ and 5′-CTAATCCGACTGATACTC-3′, for *THA1* were 5′-ATGGTGATGAGAAGTGTG-3′ and 5′-TTAGGTTCGGCTTGGTTCCTGC-3′, for *EXL2* were 5′-ATGGCTTCTAATTACCGTTTTGC-3′ and 5′-TCAAACCAGAGTCTTGCACG-3′, and for *AT1G17620* were 5′-ATGACAGACGACAGAGTTTAC-3′ and 5′-TTAGAAAGTAATTTTCCAGATC-3′. The primers for *ACT2* have been reported previously [45].

### 4.3. Constructs

The *LexA-Gal4*:*GUS* reporter, the *GD* and *LD-VP* effector, and the *NLS-RFP* nuclear indicator constructs have been reported previously [38,39].

To generate constructs for transcription activity and subcellular localization assays, the full-length open reading frame (ORF) sequences of *ASR1* and *ASR2* were amplified by RT-PCR, cloned into the *pUC19* vector under the control of the *CaMV 35S* enhancer–promoter and in frame with an N-terminal GD or a C-terminal GFP tag, respectively, as described previously [46].

To generate the *35S*:*ASR1* and *35S*:*ASR2* constructs for plant transformation, the amplified full-length ORF sequences of *ASR1* and *ASR2* were cloned into the *pUC19* vector under the control of the *CaMV 35S* enhancer–promoter and in frame with an N-terminal HA. The generated constructs of *35S*:*ASR1* and *35S*:*ASR2* in *pUC19* were then digested with the appropriate restriction enzymes and cloned into the binary vector *pPZP211* [47].

To select target sites for CRISPR/Cas9 gene editing of *ASR1* and *ASR2*, the exon sequences of *ASR1* and *ASR2* were used to identify the target sequences with the most potential on CRISPRscan (http://www.crisprscan.org/?page=sequence, accessed on 15 June 2020), and then the potential target sequences were evaluated on Cas-OFFinder (http://www.rgenome.net/cas-offinder/, accessed on 15 June 2020). Two specific target sequences were selected for each gene. The target sequences for *ASR1* were 5′- GTCCATACCACAAGAACTGTG(CGG)-3′ and 5′-GTTCCCTATCCAACGATCT(TGG)-3′, and for *ASR2* were 5′-GTCCTTACCACAAAAACTG(CGG)-3′ and 5′-CATCTCGTTCCCGATCCGA(AGG)-3′.

To generate the CRISPR/Cas9 constructs for gene editing of *ASR1* and *ASR2*, the target sequences were inserted into the *pHEE-FT* vector [48], as described for the original *pHEE401E* vector [49]. Three different constructs were generated, constructs used to generate single mutants contain the two target sequences for *ASR1* or two targets for *ASR2* as mentioned above. The construct used to generate the *asr1 asr2-c25* double mutant contains the four above mentioned target sequences. The *asr1 asr2-c2* double mutant was generated by transforming the *asr2-c23* single mutants with the construct containing two targets to *ASR1*. The primers used to introduce the target sequences into the sgRNA expression cassettes were ASR1-TG1-gRT#+: 5′-GTCCATACCACAAGAACTGGTTTTAGAGCTAGAAAT-3′, ASR1-TG1-ATU6-1T#-: 5′-CAGTTCTTGTGGTATGGACCAATCACTACTTCGTCT-3′, ASR1-TG2-gRT#+: 5′-GTTCCCTATCCAACGATCTGTTTTAGAGCTAGAAAT-3′, ASR1-TG2-ATU6-26T#-: 5′-AGATCGTTGGATAGGGAACCAATCACTACTTCGACTC-3′, ASR2-TG1-gRT#+: 5′-GTCCTTACCACAAAAACTGGTTTTAGAGCTAGAAAT-3′, ASR2-TG1-ATU6-29T#-: 5′-CAGTTTTTGTGGTAAGGACCAATCTCTTAGTCGACT-3′, ASR2-TG2-gRT#+: 5′-CATCTCGTTCCCGATCCGAGTTTTAGAGCTAGAAAT-3′, and ASR2-TG2-ATU6-1T#-: 5′-TCGGATCGGGAACGAGATGCAATCACTACTTCGTCT-3′.

### 4.4. Plant Transformation, Over-Expression Transgenic Plants and Mutants Isolation

To generate the *35S*:*ASR1* and *35S*:*ASR2* transgenic plants and gene-edited mutants for *ASR1* and *ASR2*, about 5-week-old Col wild-type Arabidopsis plants were transformed via *GV3101* Agrobacterium by using the floral dip method [50].

To isolate over-expression transgenic plants, T1 seeds were collected from constructs *35S*:*ASR1* and *35S*:*ASR2* transformed plants and germinated on 1/2 MS plates containing 50 μg/mL kanamycin and 100 μg/mL carbenicillin. T1 transgenic plants selected were planted in soil pots and grown in a growth chamber. The T2 seeds from T1 transgenic plants were collected and used to select lines with 3:1 segregation, and then homozygous T3 transgenic plants were selected from lines with 3:1 segregation. Expression levels of *ASR1* and *ASR2* in the transgenic plants were examined by RT-PCR amplification as described for ORF amplification in Section 4.2, and two independent homozygous lines of *35S*:*ASR1* and *35S*:*ASR2* transgenic plants with high expression levels were selected and used for the experiments.

To identify gene-edited transgenic plants, the T1 seeds were collected from CRISPR/Cas9 gene editing constructs transformed plants and germinated on 1/2 MS plates containing 100 μg/mL carbenicillin and 30 μg/mL hygromycin. T1 transgenic plants selected were planted in soil pots and grown in a growth chamber, and the gene editing status in T1 plants with early flowering phenotypes was examined by PCR amplifying and sequencing of the genomic sequences of *ASR1* and *ASR2*, respectively. The primers used were the same as for RT-PCR analysis. T2 seeds were collected from gene-edited T1 plants and germinated directly in soil pots and grown in a growth chamber. *Cas9*-free homozygous mutants were isolated from normal flowering T2 plants by PCR amplifying and sequencing the genomic sequences of *ASR1* and *ASR2*, respectively, and PCR amplifying of the *Cas9* fragment. The primers used for *Cas9* fragment amplification have been reported previously [48].

### 4.5. DNA Isolation

To examine the gene editing status of *ASR1* and *ASR2*, DNA was extracted from the leaves of the T1 transgenic plants and T2 progeny of the gene-edited T1 plants, and used for PCR amplification of the genomic sequences of *ASR1* and *ASR2*, respectively.

To identify *Cas9*-free gene-edited transgenic plants, DNA was extracted from the leaves of T2 progeny of the gene-edited T1 plants and used for PCR amplification of the *Cas9* fragment.

### 4.6. Plasmid DNA Isolation, Protoplasts Isolation, Transfection, GFP Observation, and GUS Activity Assays

Plasmid DNA isolation, protoplast isolation, and transfection were carried out by following the procedure described previously [13,45,51,52]. Briefly, Arabidopsis protoplasts were isolated from rosette leaves collected from ~3 to 4-week-old Col wild-type plants. Plasmid DNA was isolated by using a GoldHi EndoFree Plasmid Maxi Kit (CWBIO, Taizhou, China) by following the manufacturer’s instruction. For subcellular localization assays, plasmids of *NSL-RFP* were co-transfected with *ASR1-GFP* and *ASR2-GFP*, respectively, into the isolated protoplasts. For transcription activity assays, plasmids of *LexA-Gal4-GUS* reporter and *LD-VP* activator were co-transfected with the *GD-ASR1* and *GD-ASR2*, respectively into the isolated protoplasts. Co-transfection of *GD* was used a control for transcription activity assays.

Transfected protoplasts were incubated for 18–22 h under the dark condition at room temperature, then GFP and RFP fluorescence were examined and photos were taken under an Olympus FV1000 confocal microscope, whereas GUS activities were measured using a Synergy^TM^ HT fluorescent microplate reader (BioTEK, Winooski, VT, USA).The experiments were repeated at least twice.

### 4.7. ABA Sensitivity Assays

ABA-inhibited seed germination, cotyledon greening, and root elongation assays were performed as described previously [13,53,54]. For seed germination and cotyledon assays, seeds of the Col wild type, the *35S*:*ASR1* and *35S*:*ASR2* transgenic plants, the *asr1* and *asr2* single and the *asr1 asr2* double mutants were sterilized, generated and grown on 1/2 MS plates containing 0.5 μM ABA or solvent methanol alone as a control. Three plates were used as three replicates, every plate contained all the different plants with 30–40 seeds for each plant. The plates were kept at 4 °C for 2 days in dark condition and then transferred into the growth room. The numbers of germinated seeds were counted every 12 h, photos were taken 12 days after the transfer, and green seedlings were counted.

For root elongation assays, seeds of the Col wild type, the *35S*:*ASR1* and *35S*:*ASR2* transgenic plants, the *asr1* and *asr2* single and the *asr1 asr2* double mutants were sterilized, germinated and grown vertically on 1/2 MS plates for 3 days. Seedlings were then transferred to new 1/2 MS plates containing 10 μM ABA or control plates and grown vertically for addition 10 days, root length was measured, and percentage of inhibition was calculated. In an experiment, a total of 20 seedlings for each plant were used for the assays. The experiments were repeated at least twice.

### 4.8. Transcriptome Analysis

For transcriptome analysis, seeds of the Col wild-type, the *35S*:*ASR1* #19 transgenic plant and the *asr1 asr2–c25* double mutant were sterilized and sown into 1/2 MS plates and kept at 4 °C for 2 days. Then plates were transferred to the growth chamber. Ten-day-old seedlings were collected, frozen in liquid nitrogen and sent to the Beijing Genomics Institute (Shenzhen, China) for transcriptome sequencing and analysis. A total of 130 differentially expressed genes (DEGs) were found with decreased expression levels in the *35S*:*ASR1* transgenic plant, but increased expression levels in the *asr1 asr2* double mutant. These DEGs were subjected to gene ontology (GO) analysis under the term of biological process (https://www.arabidopsis.org/tools/bulk/go/index.jsp, accessed on 13 October 2022). Significant enrichment was defined as GO keywords with a Q value (adjusted *p* value) <0.05 being considered significantly enriched.

### 4.9. Bioinformatic Analysis

Homologs of ASRs were identified by using “protein homologs” on Phytozome (https://phytozome-next.jgi.doe.gov/, accessed on 5 February 2023). Full-length amino acid sequences of ASRs and the homologs in selected plant species were obtained on Phytozome and used for phylogenetic analysis by using “one click” mode with default settings on phylogeny (https://www.phylogeny.fr/, accessed on 7 February 2023).

## 5. Conclusions

Our results in this study show that both *ASR1* and *ASR2* are ABA response genes. We found that both ASR1 and ASR2 are serine-rich proteins and ASR1 also contains an L×L×L EAR motif, but both of them function as transcription repressors. Experiment results with over-expression transgenic plants and mutants suggest that ASR1 and ASR2 negatively regulate ABA responses in Arabidopsis in a redundant manner, and they may regulate the expression of some hormone signaling and abiotic stress response-related genes.

## Data Availability

All data are presented in the manuscript and the supplementary material.

## Supplementary Materials

The following supporting information can be downloaded at: https://www.mdpi.com/article/10.3390/plants12040852/s1, File S1: Amino acid sequences of ASRs and their homologs used for phylogenetic analysis. Table S1: Genes down-regulated in the *35S*:*ASR1 #19* transplants but up-regulated in the *asr1 asr2–c25* double mutant plants.
