# Supplementary material for: ASR1 and ASR2, Two Closely Related ABA-Induced Serine-Rich Transcription Repressors, Function Redundantly to Regulate ABA Responses in Arabidopsis
Source: Plants (Basel). 2023 Feb 14;12(4):852. doi: 10.3390/plants12040852 (PMC9959101; doi:10.3390/plants12040852)
Supplement: Supplementary file 1 [file plants-12-00852-s001.zip › File S1. Amino acid sequences of ASRs and their homologs used for phylogenetic analysis.pdf]

File S1 Amino acid sequences of ASRs and their homologs used for phylogenetic analysis.

>ASR1

MAAGSMDGIFRNIFEGCISSCDSSIQRRPYHKNCGCALHERSRGGGSATPCRHGRSEVVMFPIQRS
WSEGNSLALHLASSSSSSNLQSLSSSSSISTLASLSSTVSDIDSPI

>ASR2

MAADGIFRSIFEGCISGLDSAIERRPYHKNCGCALHDKSSGAGKNQNQRRPPSCRRHGSSESISFPI
RRSWSEGNIMAMNLFSSSSSSSNLQSLSSSSSLSNLASDLPVDNAATEEPSRSNKQLRWTIDEGED
D

>Solyc12g010400.1 (*Solanum lycopersicum*)

MATGAAGDAIFRGVFEGSISGHDLEISKRPYHRNCGCALHKSRGNCSHSSRNMNISYPIRRSWSE
GCLSLVAAASAAASGHSSPCSSPTVTVADMSKRNLLPVNEDGEDLVFRKEMKNIKRTLPR

>Solyc07g007050.1

MATGAAGDGLFRGVFDGCISGHDMGIQQRPYHRNCSCKLHKSRGNCSHSSRCTNVSYPIRRSWS
ESCLSLAAGAYAGASGHSSTCSSPASGVGGSDLTGKKNLVRSTSDEYDDVVLFKV

>Solyc07g007080.1

MATVAAEMMLQCVFDGSLSMSDMNIERRPYHKNCSCAMHKQKGENTTTCVHGRNVSFPKRQN
QKDMTLSIAASRFSSPSSSCNNSVVTSV

>Glyma.09G094900.1 (*Glycine max*)

MASAAAADGLFRPIYEGCISAYDNDVERRPYHKNCGCALHSKSRRNSSRACRHKLPKCNNVSYP
MRRAWSEGNLSMVSSTTSTHSSPSSSPAAGFRPQHDEEGNTKNKLVVLFEMNN

>Glyma.15G202600.1

MAAAADGLFRPIYEGCISAYDNDVERRPYHKNCGCALHSKSRRNSRACTHKLPKCNNVSYPMR
RAWSEGSLSMASATTSAHSSPSSSPAAGFRPQHDEEGNSNKLGVLFEM

>Glyma.13G157000.1

MATGATPDGLFWSVYEGCISCYDNCVERRPYHRNCGCALHNKSLINCTHKLPRCNNVSYPMRR
AWSEGSLVLAASSSSPSQVATGGRPQPSLANVEEENKNCFS

>Glyma.17G104600.1

MATGAAPDGLFWSVVYEGCISGYDNCVERRPYHRNCGCALHNTSLINRTHKLPRCNNVSYPMR
RAWSEGSLVLATSSSSSSPSQVATGGRPQPSLANVEEENKNCFNY

>Glyma.17G104700.1

MATGACQMMFQCVFEGSISLHDMEIERRPYHKNCGCALHNLNDGICSKACPQQRYVSFSKKTS
WTDYCMHTTASKFSSHLSFLSKTRH

>Glyma.13G157100.1

MATGACQMMFQCVFEGSISLHDMEIERRPYHKNCGCALHNLNDGICSKACPQQRYISFRKKTSW
TDCCMHTTASKFYSRSFLSKTRQ

>Potri.018G113700.1 (*Populus trichocarpa*)

MATGSADGFFRHVYDGCLSSGDMGIDRRPYHRNCRCALHKSKENCPHALPRCKNVSYPIKRSW
SEGSLALMIANSSSSSCHSSPSSSPSLQAGKSTSTPSHQRRLSHDLEDKLAL

>Potri.006G189600.1

MAAGAADGFFRYVHDGCLSGGEMGIDRRPYHRNCSCALHKSKENCSHAMSRYKNVSYPIKRC
WSEGSLALMVANSSSCCHSPPSSSPSLQAGKSTATPHQRRLSHDLDLEDQVCSFKV

>Potri.018G113900.1

MFIAASDIMFRCVFDGCISVDNMEIERRPYHRNCSCALHKMEGGSSTGFPLPRNMFYPKKQSWR
RCSLSLATPSILLAYQLKVNSFSYTNVNVLIFVFQKYILFMKFLFVSNLQYEIQKKTRS

>Potri.006G189800.1

MFSAASDIMFRCVFDECISMDSMETERRPYHRNCNCALHKSKAGSSTSFPQPRNILFPKKQSWRD
CSLSLVTYQPRVKQILAGPCYKWQPCLQAKK

>Potri.006G189700.1

MATGAAEMILKCIFSGSISLNDMEIRRRPYHRNCKCALHRLKDICSDACPKQRSISFPKKQVRSDH
PLSIATSRLSSPSSTADDSSMQNIGSLFASETAITDNLN

>Dioal.03G029400 (*Dioscorea alata*)

MEAAQGLLQCVLDGCLSGFDDEIRRRPYHRNCSCALHRSEDKAHAHHCFASSKISFPLRGSWYH
ASISYSLSMDYVRS

>GSMUA_Achr5G22870_001 (*Musa acuminata*)

MAAGAEGWIRCVLDGCISAFDSEIRRRPYHRNCSCALHKFRCSSRHDPCHAKISYPISLSSERLRIT
MPSSSSSSSSCFTKAASDHGRGKIEEEPWHLLQALEHFRCVGC
